# Empowering EVAR: Revolutionizing Patient Understanding and Qualification with 3D Printing

**DOI:** 10.3390/jcdd11110365

**Published:** 2024-11-10

**Authors:** Michał Kargul, Patryk Skórka, Piotr Gutowski, Arkadiusz Kazimierczak, Paweł Rynio

**Affiliations:** Department of Vascular Surgery, Pomeranian Medical University in Szczecin, Al. Powstańców Wielkopolskich 72, 70-111 Szczecin, Poland

**Keywords:** endovascular aneurysm repair (EVAR), patient education, 3D printing, preoperative education

## Abstract

Background: This study addresses the need for enhanced patient education in the context of abdominal aortic aneurysm (AAA) and its treatment through endovascular aneurysm repair (EVAR). Effective patient education is essential for improving comprehension and engagement, particularly for those facing complex medical conditions. Methods: A total of 55 patients scheduled for EVAR participated in the study. Patient-specific three-dimensional (3D)-printed models of the participants’ aneurysms were created using computed tomography angiogram (CTA) scans. The educational intervention included a structured session utilizing these 3D models, with assessments conducted before and after the session, including the Mini-Mental State Examination (MMSE). Statistical analyses evaluated the knowledge gain and its correlation with cognitive function. Results: The results showed a significant increase in knowledge scores post-education (*p* < 0.001), with a mean knowledge gain of 2.36 points. Patients rated the effectiveness of the 3D models highly on a Likert scale, with a mean score of 4.64 for improving their understanding of the medical condition and procedural aspects. A weak correlation was observed between MMSE scores and knowledge test results. Conclusions: This study demonstrates the potential of patient-specific 3D models to enhance patient education in the context of EVAR, improving patients’ understanding of their medical condition and the procedure and thereby facilitating more informed decision-making.

## 1. Introduction

An abdominal aortic aneurysm (AAA) is a critical dilation of the aorta in the abdominal region, representing a life-threatening condition that can be treated through either open surgical repair (OSR) or endovascular repair (EVAR). The endovascular approach is increasingly being utilized not only for the treatment of abdominal aortic aneurysms but also for managing pathologies involving the descending aorta and aortic arch [1,2]. To ensure the delivery of the highest quality of care, it is imperative that patients are well informed and actively involved in their healthcare journey [3]. A pivotal aspect in enhancing the quality of care is physicians’ comprehension of their patients’ unique needs and preferences [4]. However, patients, especially those who are elderly or experiencing anxiety, often struggle with retaining and accurately recalling medical information [5]. To address this challenge, it is recommended to present information concisely and in a clear, straightforward manner. Additionally, the incorporation of visual communication aids has proven particularly effective in aiding patients with lower literacy levels [3]. Physicians can employ a rating scale to gauge patients’ comprehension of medical information while remaining attentive to the potential gap between perceived and actual understanding [6].

Within the realm of EVAR, a critical diagnostic tool employed is the computed tomography angiogram (CTA), which provides detailed imaging of the patient’s abdominal and pelvic region. This imaging modality is utilized to generate intricate virtual three-dimensional (3D) representations, significantly aiding surgeons in their decision-making processes. These highly detailed 3D images serve as invaluable assets, primarily during preoperative consultations. They allow healthcare providers to elucidate the intricate nuances of the procedure and comprehensively convey the associated risks to their patients.

The advent of 3D printing technology has introduced a novel dimension to the field. It has the potential to revolutionize the interaction between patients and healthcare professionals, particularly in the context of EVAR. This groundbreaking technology enables the creation of patient-specific 3D-printed models, precisely mirroring an individual’s unique anatomical features. These patient-specific models hold significant promise as educational aids, particularly when explicating complex medical procedures such as EVAR and the associated risks involved.

By employing these patient-specific 3D-printed models, healthcare providers can navigate a new era of patient education, promoting a profound understanding of the medical condition and the intricacies of the proposed intervention. Such models act as tangible, tactile tools, facilitating effective communication between patients and healthcare professionals, transcending traditional limitations of 2D images and medical jargon. In the context of EVAR, patient-specific 3D models have the potential to not only bridge the communication divide but also empower patients by providing them with a tangible representation of their unique pathology. This hands-on approach to education can significantly enhance patients’ comprehension, allowing them to make more informed decisions about their healthcare.

Three-dimensional-printed models have emerged as versatile tools, playing a pivotal role beyond surgical planning and medical education [7,8,9,10,11]. However, it is worth noting that studies employing such models in patient education remain relatively scarce [12]. Nevertheless, it is important to underscore that the potential benefits were assessed by physicians and not the patients themselves.

Given the promising potential of 3D-printed AAA models as a tool for patient education, this study aims to assess the role of patient-specific 3D AAA models in enhancing patient education and explore the potential correlation between the Mini-Mental State Examination (MMSE) test results in patients and the effectiveness of 3D patient-specific models as educational aids.

## 2. Materials and Methods

### 2.1. Study Design and Setting

This research was a single-center, prospective, pre–post intervention study designed to assess the efficacy of patient-specific 3D AAA models in educating patients undergoing EVAR. The study was carried out at the Department of Vascular Surgery, Pomeranian Medical University in Szczecin spanning from November 2022 to January 2023.

### 2.2. Study Population and Sampling

The study cohort comprised patients scheduled for EVAR due to infrarenal AAA. Inclusion criteria encompassed individuals aged 18 years or older, a confirmed AAA diagnosis via CTA, and the ability to provide informed consent. Exclusion criteria encompassed severe cognitive impairment, prior exposure to 3D-printed AAA models, and a medical history positive for an EVAR or TEVAR procedure. A total of 55 eligible patients consented to participate in the study.

### 2.3. Preparation for a 3D-Printed Model

Each enrolled patient underwent the creation of a patient-specific, one-to-one scale 3D model based on CTA images taken before the EVAR procedure, utilizing various CTA scanners. The 3D modeling process encompassed the mesenteric artery, celiac trunk, both renal arteries, AAA, and common iliac arteries. Segmentation and modeling were executed using open-source software, 3DSlicer 5.3.0 [11], where vessel data were extracted through a combination of thresholding and manual removal of non-vascular data. Subsequently, a model of the surface of AAA was derived from the vessel data of the CTA. The resulting STL models were processed through the 3D Gence slicer (3D Gence, Pyszkowice, Poland) and were printed using a 3D Gence F340 printer (3D Gence, Pyszkowice, Poland). The model was constructed using Acrylonitrile Butadiene Styrene (ABS) plastic with High-Impact Polystyrene (HIPS) supports. The segmentation team consisted of three members: one physician and two students who work in the vascular surgery department and in-hospital 3D-printing laboratory. Prior training on Horos 3.0 software (Horos Project, Annapolis, MD, USA) and 3DSlicer 5.3.0 [11] was provided to the team, who had previous experience in performing AAA segmentations and 3D printing.

### 2.4. Intervention

The educational session took place on the day preceding the EVAR procedure, held in a dedicated, distraction-free environment. The session, delivered by a trained researcher adhering to a standardized script and performed by the same operator, spanned approximately 15–20 min.

### 2.5. Data Collection and Analysis

Data collection consisted of two phases: pre-education and post-education. In the pre-education stage, following conventional information presented by a vascular surgeon, patients completed a demographic questionnaire, providing details on their age, gender, educational background, occupation, and comorbidities. Additionally, patients filled out a customized questionnaire (yes—1 point, or no—0 points) to gauge their knowledge concerning EVAR and AAA. The questionnaire can be found in the Supplementary Materials. Using a Likert scale from 1 (not at all) to 5 (very much), patients rated their understanding of the disease and procedure, the extent to which the 3D-printed model facilitated comprehension of the procedure and disease, and assessed the quality of the provided information. Patients were also asked to self-assess their knowledge before and after the 3D model presentation. In the post-education phase, the same structured approach was maintained. However, a key distinction lay in the fact that patients were afforded the opportunity to engage with the 3D model directly. The MMSE was administered as part of the comprehensive assessment protocol in this study. The MMSE is a widely accepted tool for evaluating cognitive function and was employed to measure the participants’ cognitive status. The administration of the MMSE was carried out by a previously trained team, which was also responsible for segmentation and 3D printing. Those individuals received training under the supervision of a qualified neurologist to ensure adherence to standardized procedures and professional conduct in line with medical guidelines. The MMSE was conducted before the educational intervention to gauge any potential impact on cognitive function associated with the intervention.

### 2.6. Statistical Analysis

The data analysis was performed using Statistica 13.3 (StatSoft Polska Sp. z o.o., Kraków, Poland). Descriptive statistics were used to summarize the demographic characteristics and knowledge scores of the group. Discrete numbers were used to present the frequencies of comorbidities. The questionnaire data ranked on the Likert scale as well as the knowledge test results were presented as the mean and standard deviation. The normality of distribution was checked by interpreting the histograms and using the Shapiro–Wilk test. Wilcoxon tests were used to compare the pre-education and post-education knowledge scores within the 3D model group. The assessment of correlations between the knowledge test scores and the results of the MMSE before and after the educational intervention, as well as the examination of the correlation between knowledge gain and MMSE test results, was conducted utilizing the Spearman rank correlation test. The level of significance was set at *p* < 0.05.

## 3. Results

### 3.1. Patients’ Cohort

The study sample comprised 55 patients who met the inclusion criteria. Among them, 47 individuals (85.45%) were male, while 8 individuals (14.55%) were female. The mean age of the participants was 71 ± 6.99 years, with a range from 52 to 89 years. Smoking data indicated that the mean number of packs smoked per year was 21.55 ± 18.91, with a median of 19. The mean number of points accumulated in the MMSE test by patients was 27.81 ± 2.43, with a median of 28 points.

**Figure 1:**
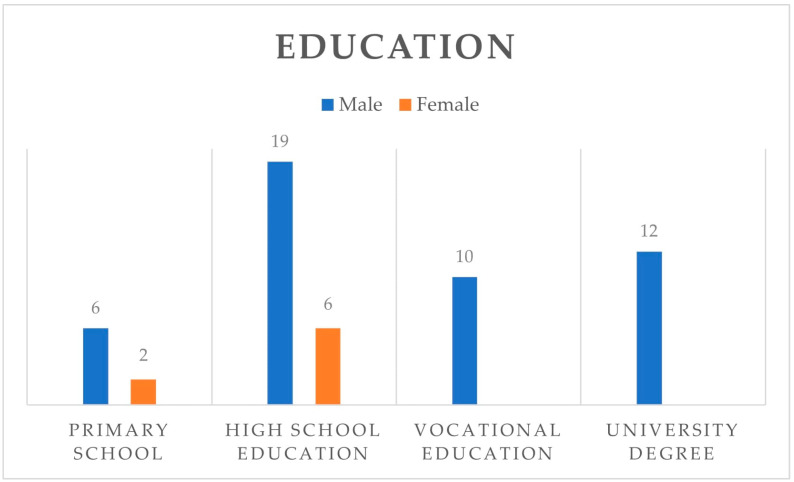
The educational background of patients.

The complete frequencies of comorbidities and previous surgeries within the patient population can be found in Table 1. The educational background of the patients is presented in Figure 1. A summary of the frequency of responses from patients to each question before and after the educational session is presented in Table 2.

### 3.2. Results of Education Through 3D Model

**Figure 2:**
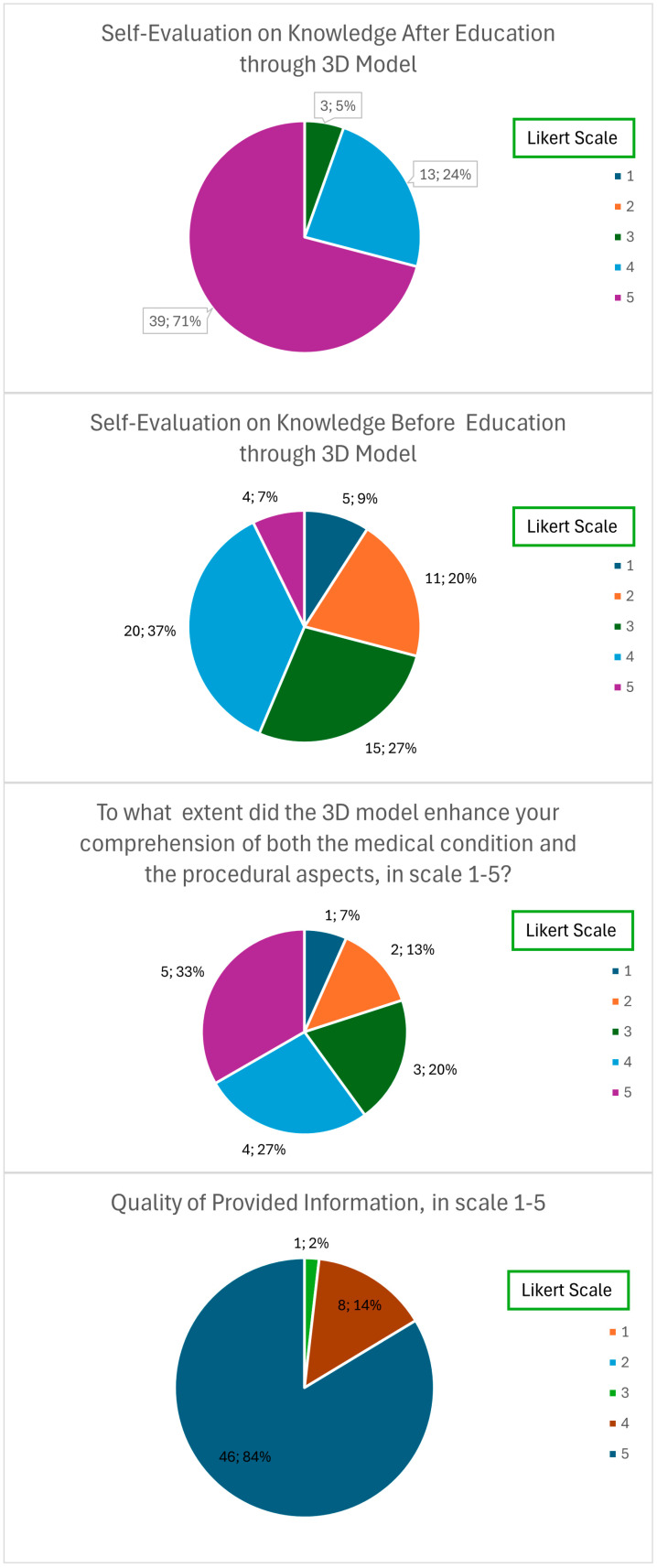
Individual responses (number of responses and percentage value).

Prior to the educational session utilizing the 3D model, the mean self-evaluation score was 3.13 ± 1.10, with a median score of 3. Individual responses are provided in Figure 2.

After the educational intervention, the score exhibited a statistically significant increase, reaching 4.65 ± 0.58, with a median score of 5 (*p* < 0.001). Detailed individual responses are presented in Figure 2.

Utilizing a Likert scale ranging from 1 to 5, patients assessed the effectiveness of the 3D model in enhancing their understanding of both the medical condition and the procedural aspects. The mean score was 4.64, with both the median and mode being 5. The distribution of individual responses is depicted in Figure 2.

The mean score on the customized questionnaire test before the educational intervention was 6.13 ± 2.12, and after the intervention, it increased to 8.49 ± 1.17, with statistical significance at *p* < 0.001. The mean knowledge gain in points was 2.36 ± 1.66.

Utilizing a Likert scale ranging from 1 to 5, patients assessed the quality of information provided with the use of a 3D-printed model, with a mean score of 4.82 ± 0.43 with both the median and mode being 5. The distribution of individual responses is depicted in Figure 2.

### 3.3. Correlation Between MMSE Test and Education with 3D Model

The test result before the presentation of the model weakly correlates with the result of the MMSE test (R = 0.343229; *p* = 0.01) according to the Spearman rank test. The post-model presentation test result exhibits a modest correlation with the outcome of the MMSE test (R = 0.38; *p* = 0.005) as determined by the Spearman rank test. In our analysis, knowledge gain did not exhibit a significant correlation with the MMSE test results (r = −0.23), with the *p*-value indicating a lack of statistical significance (*p* = 0.09).

## 4. Discussion

### 4.1. Role of This Study

The role of this study was to evaluate the educational impact of 3D models on patients, enhance their comprehension of medical conditions and procedures, and determine whether 3D models could be a valuable tool for patient education in the context of vascular surgery.

In accordance with precedent inquiries, notably the Eisenmenger project, our study corroborates the assertion that the integration of 3D models significantly enhances patients’ understanding of their medical condition, specifically in the realm of AAA [12]. The parallelism between the studies is evident in the application of printed patient-specific 3D models and a congruent methodology. Notably, while our investigation focused on AAA, the Eisenmenger project encompassed both thoracic endovascular aortic repair and EVAR patients, providing a broader scope to their evaluation.

This investigation diverges from the research spearheaded by Khural et al. in terms of its primary focus, methodological approach, and resultant findings [13]. In contrast to the emphasis of Khural et al. on the evaluation of the physical attributes of a 3D-printed model with expert medical input, our study was designed to appraise the influence of specific 3D models on patients’ comprehension and procedural knowledge. Notably, we observed that even seasoned physicians acknowledged the augmented value of employing 3D-printed models in enhancing patients’ preoperative education [13].

One noteworthy outcome of our study is the substantial knowledge gain observed among patients who received education with the assistance of 3D models. The mean knowledge gain amounted to 2.36 points, signifying a statistically significant enhancement in patients’ comprehension of AAA and the EVAR procedure. This finding underscores the value of 3D models as effective tools for patient education, facilitating substantial knowledge acquisition within a relatively brief educational session.

In conclusion, our study highlights the considerable potential of 3D-printed AAA models in patient education, enriching patients’ comprehension of their condition and associated procedures. These findings advocate for the broader integration of 3D models into the patient education process, potentially fostering more well-informed and empowered patients within the realm of vascular surgery. However, further studies involving larger cohorts and long-term follow-ups are warranted to validate and expand upon our findings.

### 4.2. The Role of Patient Education and Effective Doctor–Patient Communication in Healthcare

The significance of well-informed and engaged patients within the healthcare domain cannot be overstated. Informed medical consent is the cornerstone of the patient–physician relationship, necessitating patients’ active participation in comprehending the risk–benefit dynamics associated with proposed treatment strategies [3,14,15,16,17].

Prior research has consistently demonstrated that patient education can yield multiple benefits, including enhanced patient satisfaction, improved compliance, better quality of life, anxiety reduction, decreased readmissions, and a reduction in costly medical errors [18]. These findings align with previous studies, which have reported increased satisfaction among better-informed patients [19]. Furthermore, the review of the literature underscores the anxiety-reduction effect of preoperative patient education in individuals scheduled for various surgical procedures [20].

Effective communication between doctors and patients is the cornerstone of a robust therapeutic relationship. As per the insights from the review, proficient doctor–patient communication can assist in regulating patients’ emotions, facilitating the comprehension of medical information, and identifying patients’ needs and perceptions [21]. It is imperative that patient education methods be tailored to individuals’ needs, encompassing various educational modalities such as written materials, videotapes, audiotapes, verbal instruction, demonstration, and mixed-reality approaches. As noted in the review, when employing written educational materials, numerous factors, including content, language, organization, layout, illustration, learning, and motivation, must be meticulously considered to maximize their efficacy [22]. Preoperative patient education plays a pivotal role in this context, and our study illuminates how 3D-printed models can significantly enhance patients’ understanding. The employment of a Likert scale-based assessment revealed notably high patient ratings for the 3D models, with an average score of 4.64 on a scale of 1 to 5. This underscores the profound utility of 3D models in elucidating complex medical conditions and procedures.

### 4.3. Limitations

This research encompasses several noteworthy limitations that necessitate thoughtful consideration. Firstly, the sample size, consisting of 55 patients, although yielding encouraging results, remains relatively modest in scale. A more expansive and diverse cohort would be imperative to enhance the broader generalizability of the findings. Secondly, the study’s confinement to a single medical center raises potential constraints on the extent to which the results can be extrapolated to a more comprehensive population. Multicenter studies, encompassing diverse healthcare settings, could provide a more panoramic view of the subject matter.

Furthermore, the voluntary nature of patient participation introduces the potential for selection bias, as those who chose to engage in the study may possess distinct characteristics or motivations compared to those who declined to participate, potentially affecting the overall representativeness of the sample. Moreover, the study primarily concentrated on short-term outcomes immediately following the educational session, with limited exploration of the sustainability of knowledge retention over the long term and the lasting impact of the educational intervention.

Additionally, the reliance on patients’ self-assessment of their knowledge, although a valuable component of the study, may not consistently align with their actual comprehension due to its susceptibility to personal interpretation and bias. Finally, the study employed the MMSE as a measure of cognitive function. While this tool enjoys widespread recognition, it harbors inherent limitations and may not comprehensively capture the full spectrum of cognitive abilities among all patients. These limitations, although present, should not diminish the valuable insights and contributions of this research but rather serve as a foundation for further inquiry and refinement in future studies.

## 5. Conclusions

The utilization of 3D-printed patient-specific models notably enhanced patients’ comprehension of their medical condition and knowledge pertaining to the procedural aspects. Intriguingly, the observed knowledge gain did not exhibit a correlation with the MMSE scores. This signifies that the efficacy of 3D models in facilitating knowledge acquisition extends across patients with varying cognitive profiles, including those with dementia. The study suggests that specific 3D models serve as an effective educational tool, transcending cognitive boundaries and catering to individuals both with and without cognitive impairment.

## Figures and Tables

**Table 1:** Comorbidities and Previous Surgeries.

Disease/Surgery History	Number of Patients	%
hypertension	43	78.18
diabetes mellitus type 2	7	12.73
asthma	2	3.64
chronic obstructive airway disease	2	3.64
peripheral artery disease	11	20.00
ischemic heart disease	21	38.18
TIA ^1^/stroke	7	12.73
chronic kidney disease	5	9.09
atrial fibrillation	9	16.36
CABG ^2^	5	9.09
PTCA ^3^	8	14.55

^1^ transient ischemic attack; ^2^ coronary artery bypass grafting; ^3^ percutaneous coronary angioplasty.

**Table 2:** Frequency of Responses to Each Question Before and After the Educational Session.

	Before Intervention (*n* = 55)		After Intervention (*n* = 55)	
Questions	YES	NO	YES	NO
What procedure will be performed?	45	10	53	2
What is the aorta?	34	21	50	5
What is an aneurysm?	43	12	50	5
What are the symptoms of an aneurysm?	26	29	53	2
What is a stent graft?	27	28	51	4
In which area of the body will the procedure be conducted?	42	13	53	2
How is a stent graft implanted?	24	31	48	7
What are the consequences of an untreated aneurysm?	51	4	55	0
Why is a stentgraft implanted?	45	10	54	1
Should information about the procedure be provided with a 3D model?	30	25	54	1

## Data Availability

The data that support the findings of this study are available from the corresponding author upon reasonable request.

## Supplementary Materials

The following supporting information can be downloaded at https://www.mdpi.com/article/10.3390/jcdd11110365/s1.
