# Supplementary figures and images for: Empowering EVAR: Revolutionizing Patient Understanding and Qualification with 3D Printing
Source: J Cardiovasc Dev Dis. 2024 Nov 10;11(11):365. doi: 10.3390/jcdd11110365 (PMC11594954; doi:10.3390/jcdd11110365)

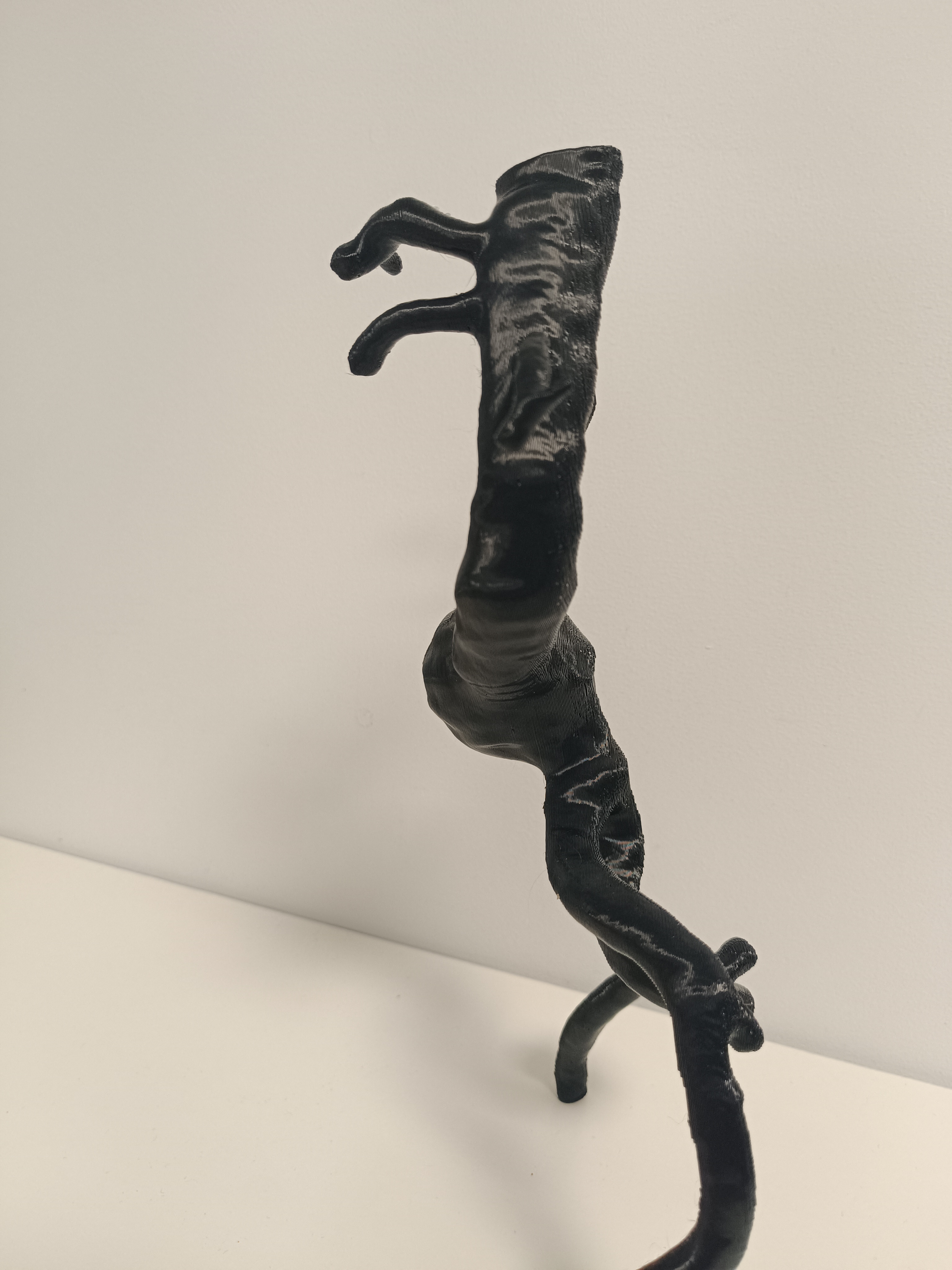

Supplement: Supplementary file 1 [file jcdd-11-00365-s001.zip › 1727619774213.jpg]

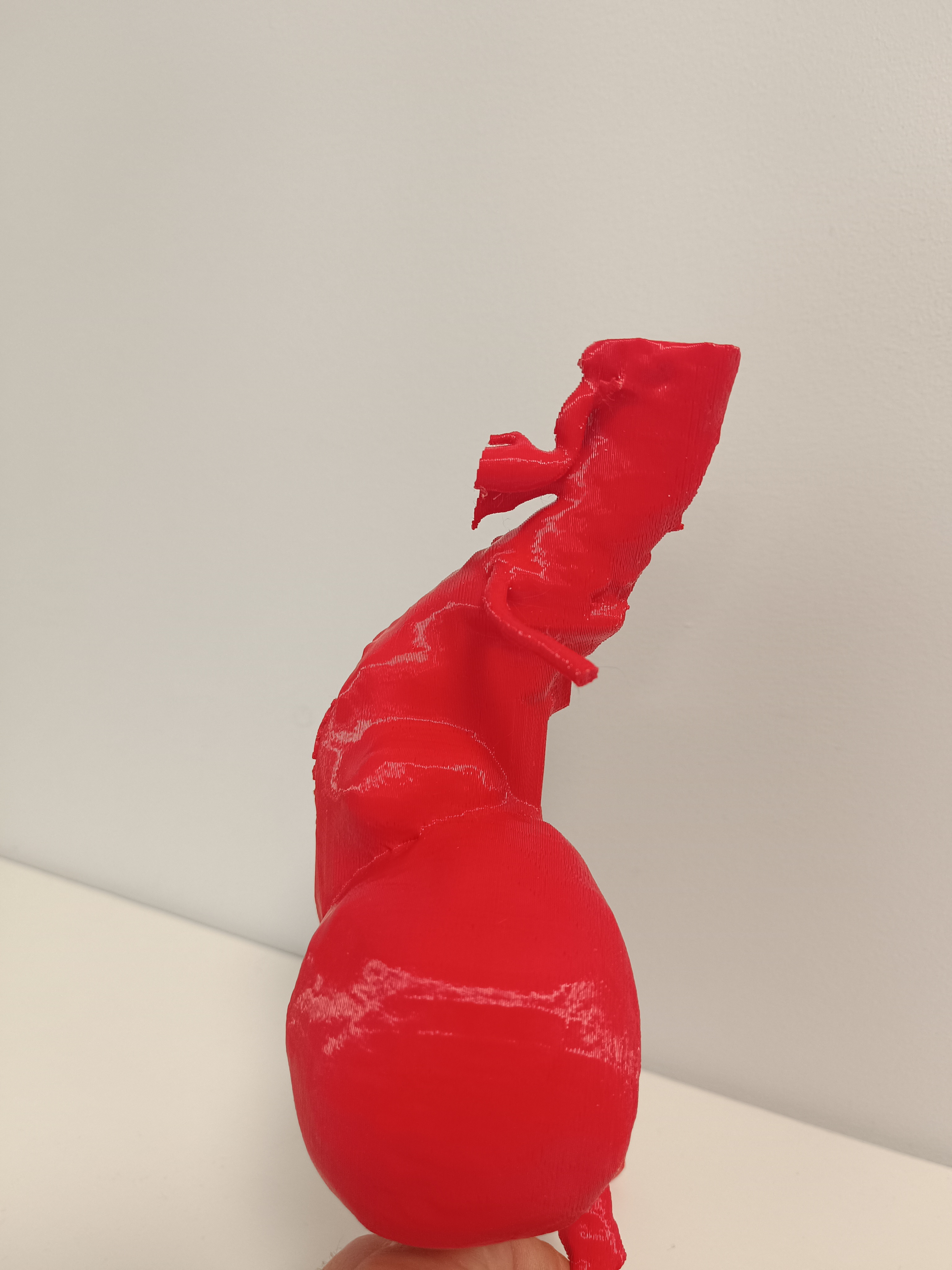

Supplement: Supplementary file 1 [file jcdd-11-00365-s001.zip › 1727619774197.jpg]

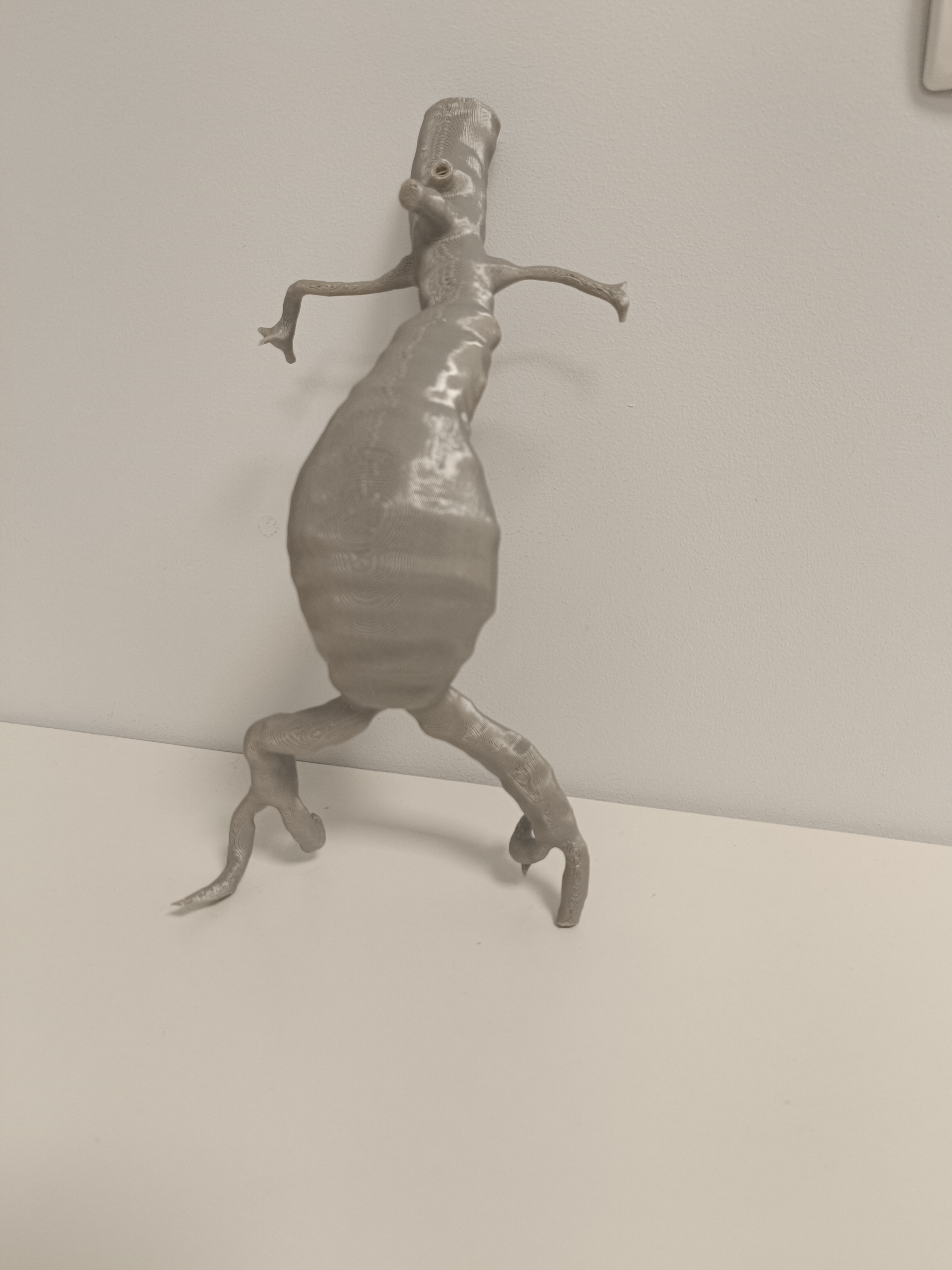

Supplement: Supplementary file 1 [file jcdd-11-00365-s001.zip › 1727619774187.jpg]

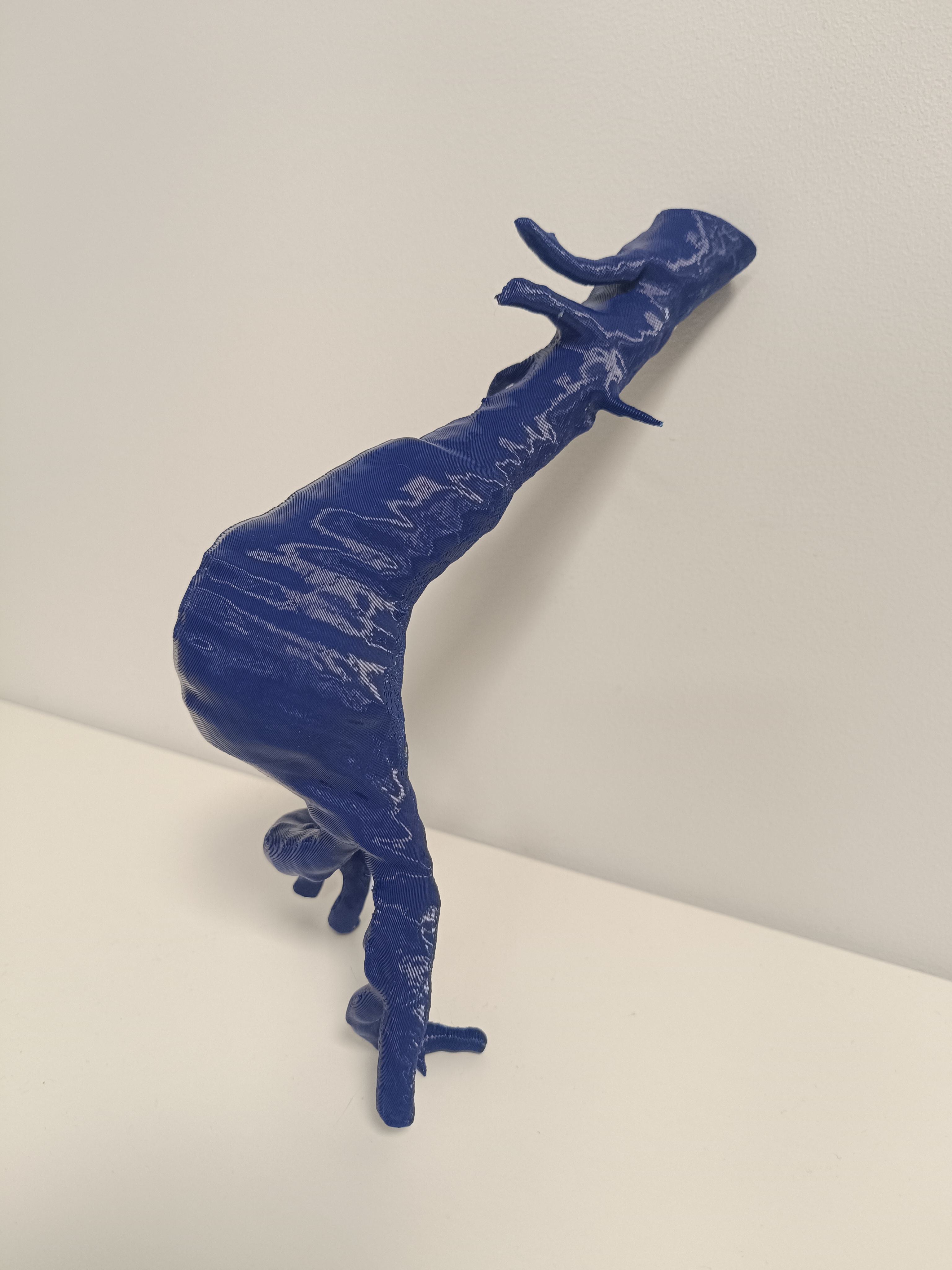

Supplement: Supplementary file 1 [file jcdd-11-00365-s001.zip › 1727619774191.jpg]

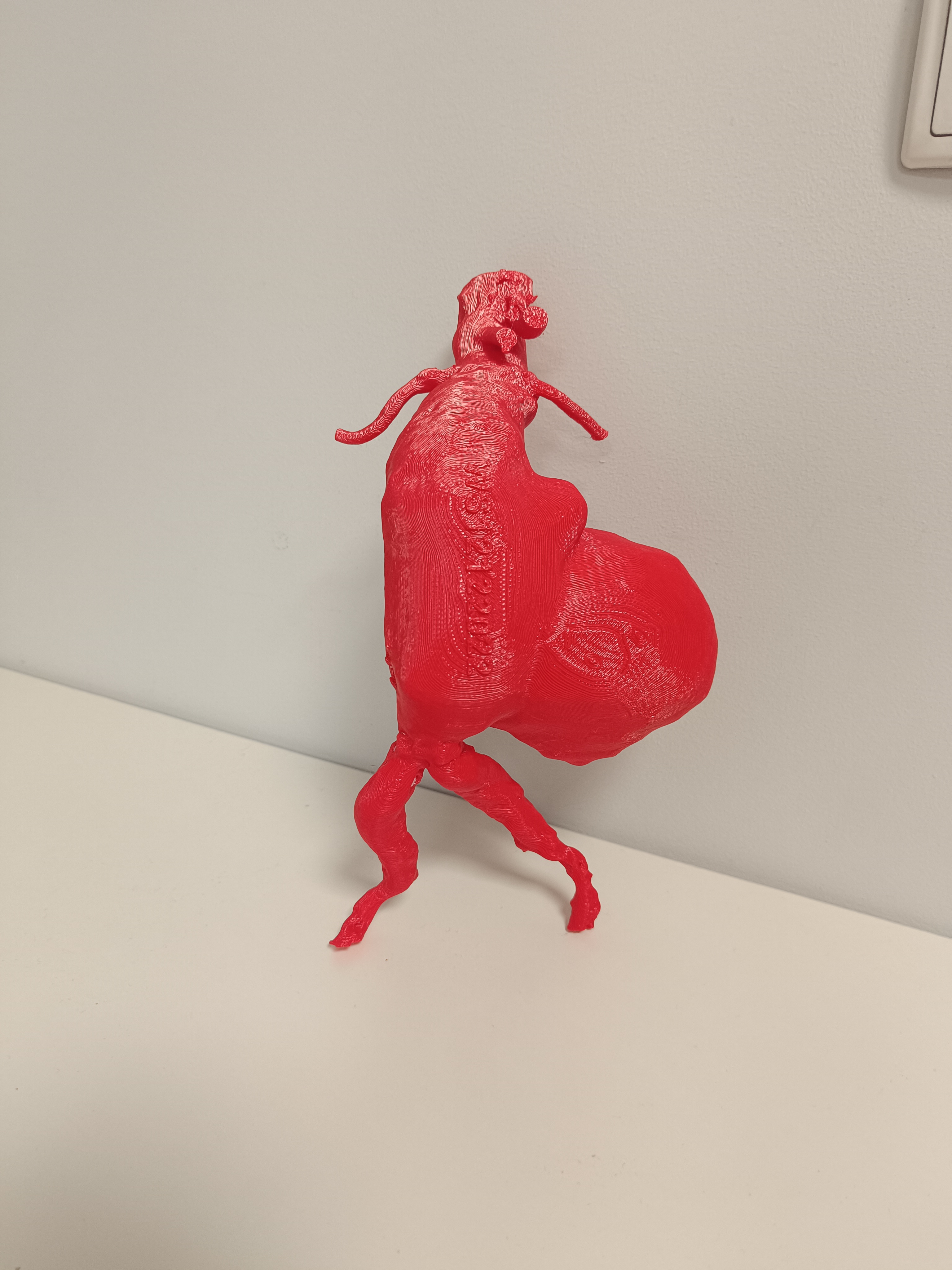

Supplement: Supplementary file 1 [file jcdd-11-00365-s001.zip › 1727619774204.jpg]

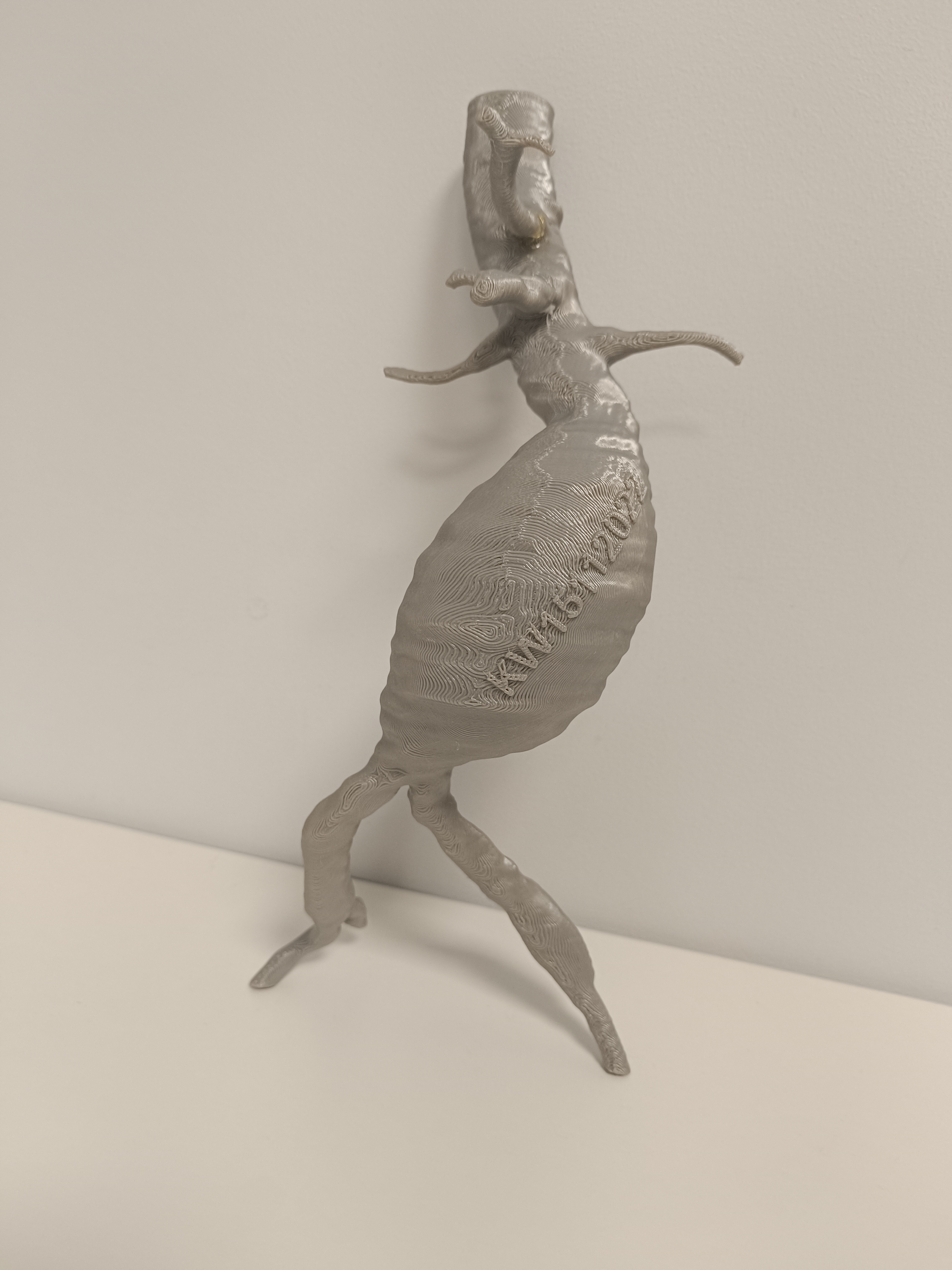

Supplement: Supplementary file 1 [file jcdd-11-00365-s001.zip › 1727619774167.jpg]

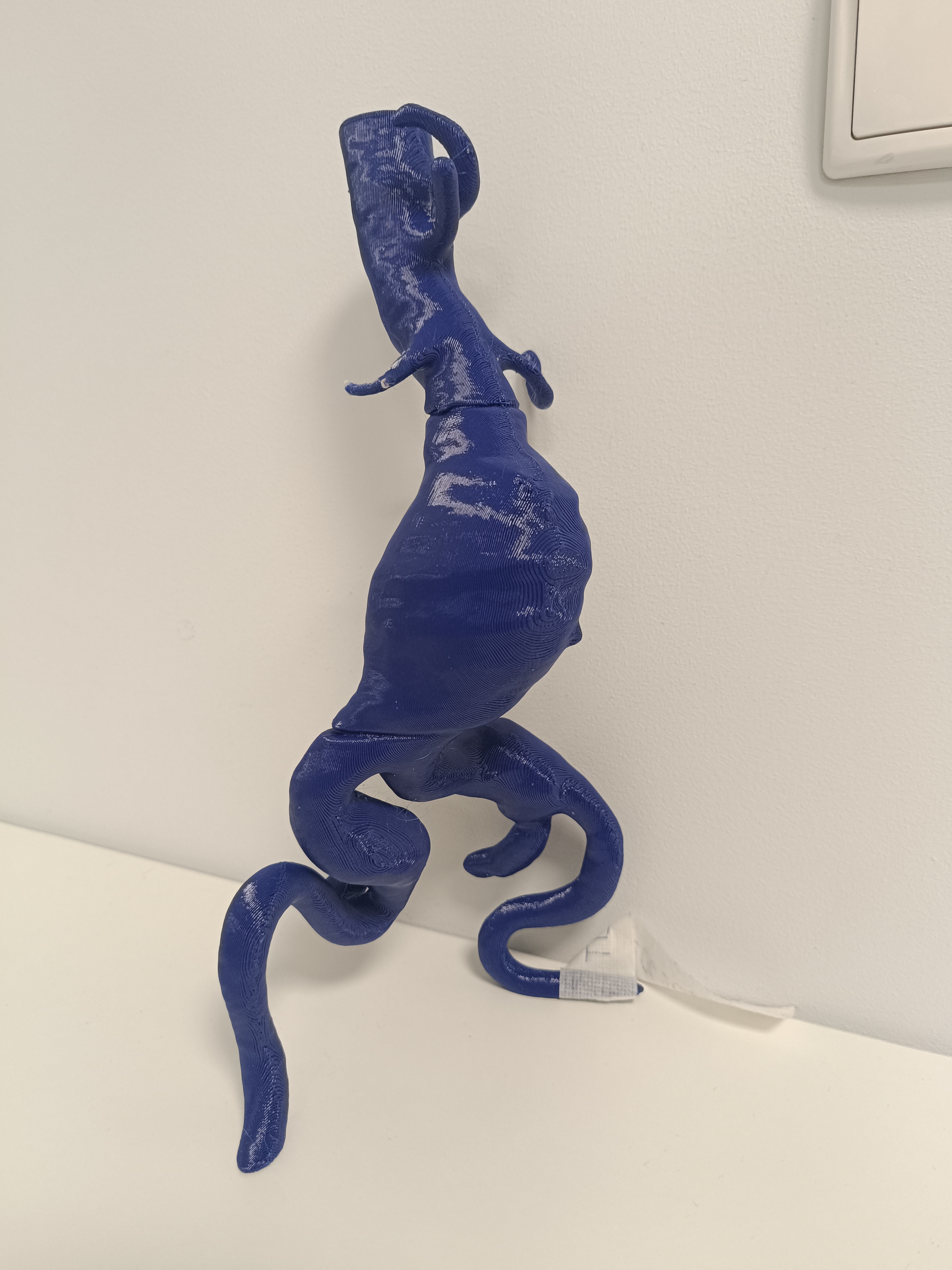

Supplement: Supplementary file 1 [file jcdd-11-00365-s001.zip › 1727619774170.jpg]

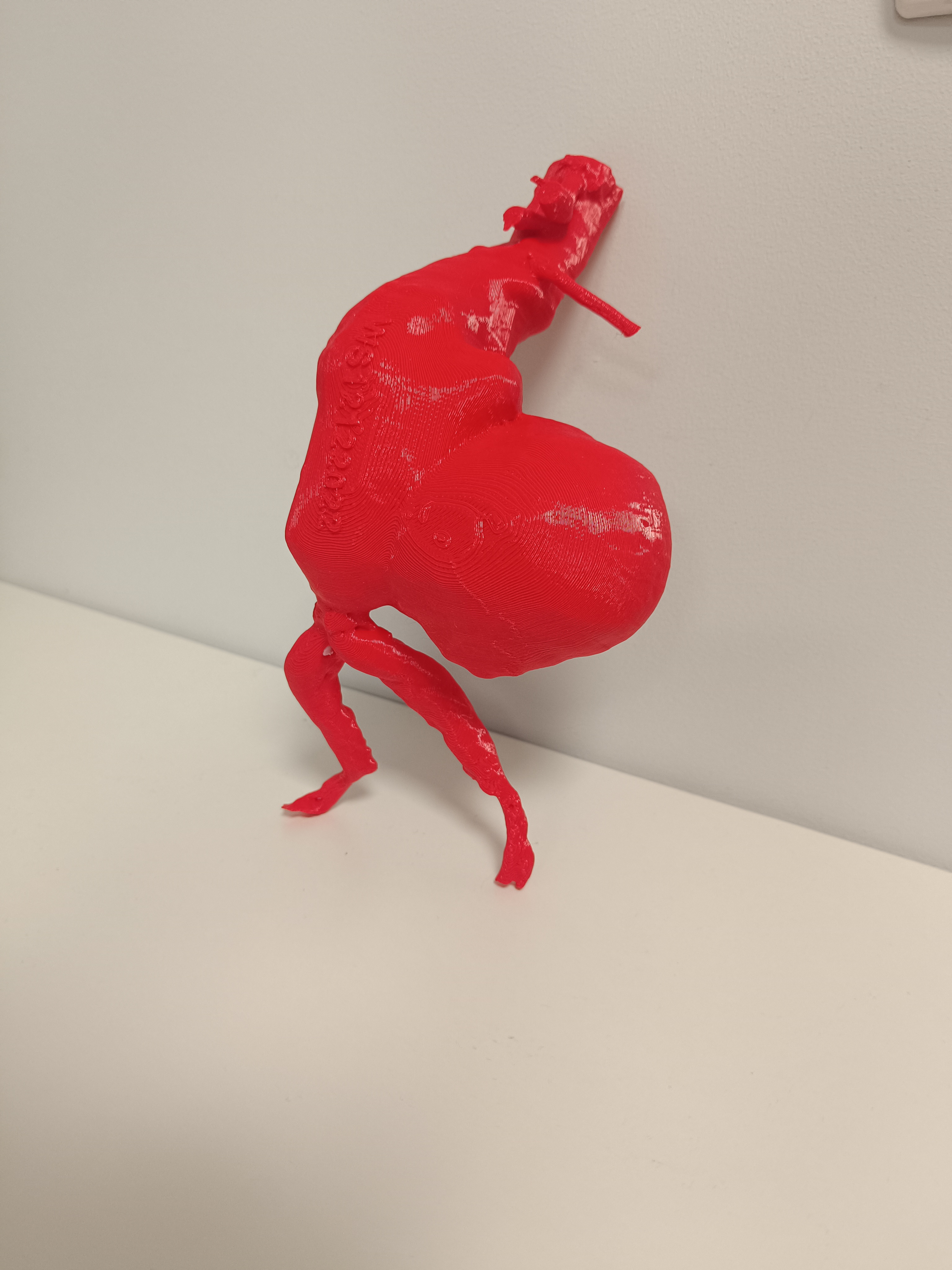

Supplement: Supplementary file 1 [file jcdd-11-00365-s001.zip › 1727619774209.jpg]

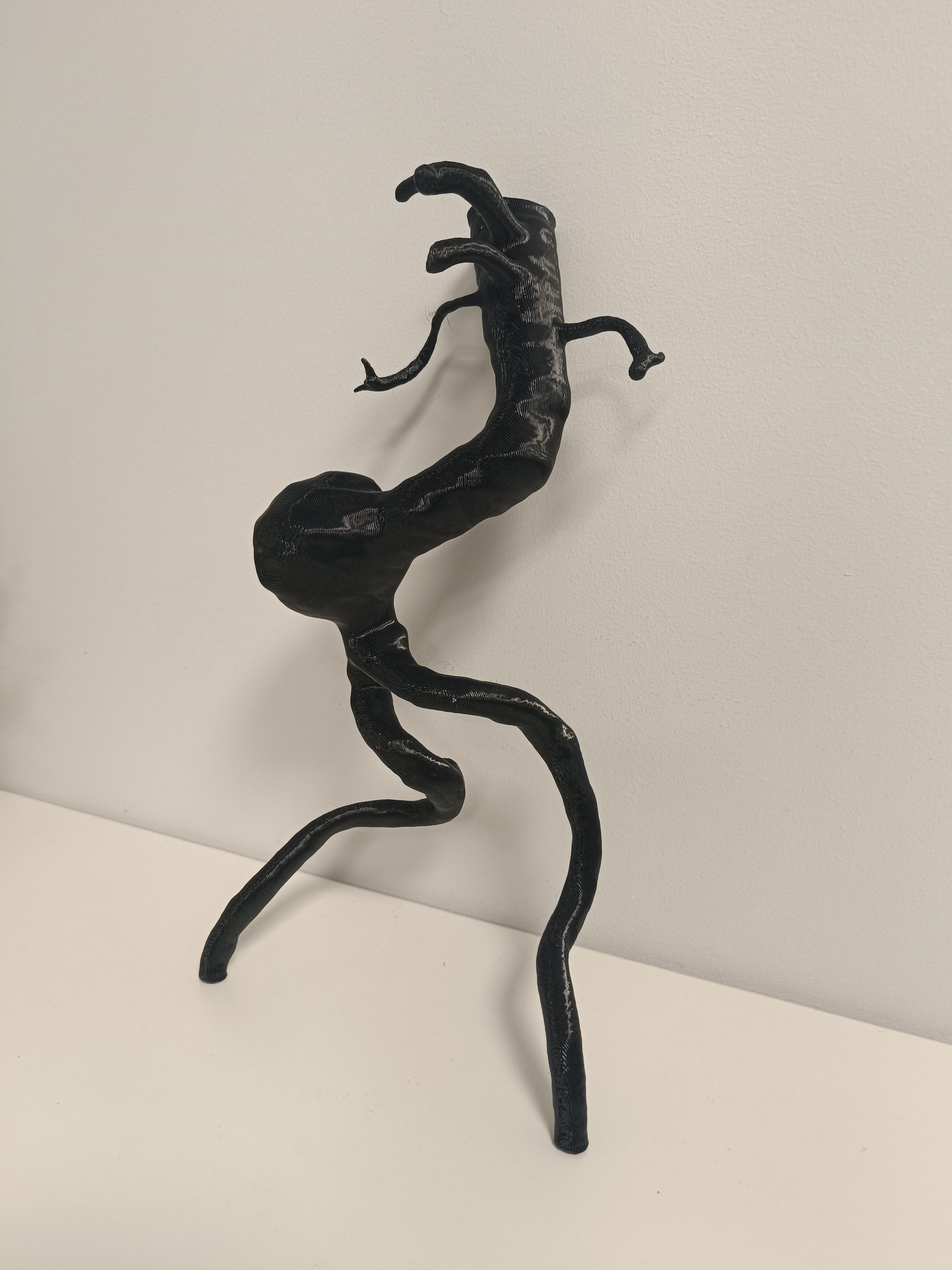

Supplement: Supplementary file 1 [file jcdd-11-00365-s001.zip › 1727619774233.jpg]

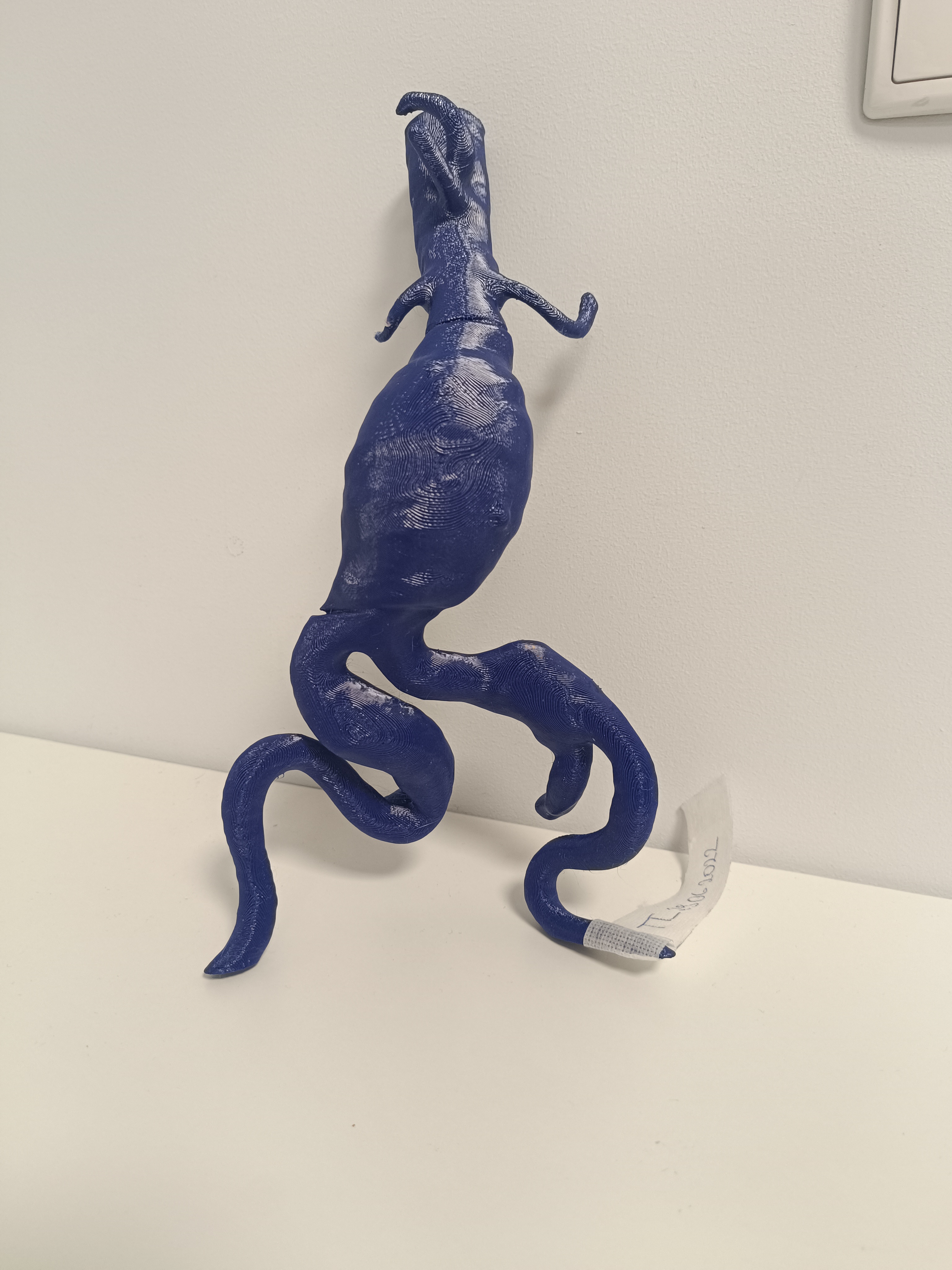

Supplement: Supplementary file 1 [file jcdd-11-00365-s001.zip › 1727619774178.jpg]

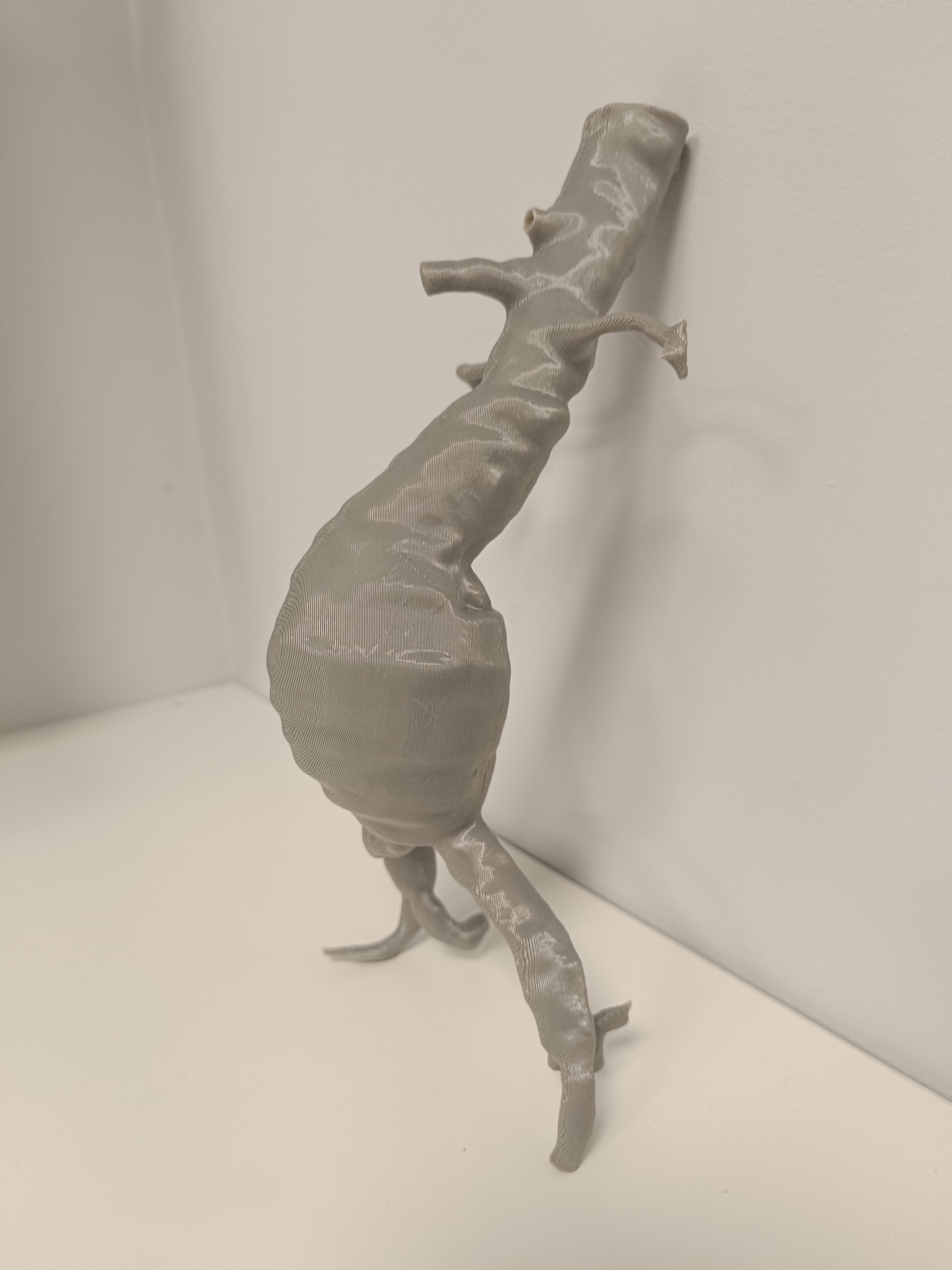

Supplement: Supplementary file 1 [file jcdd-11-00365-s001.zip › 1727619774184.jpg]

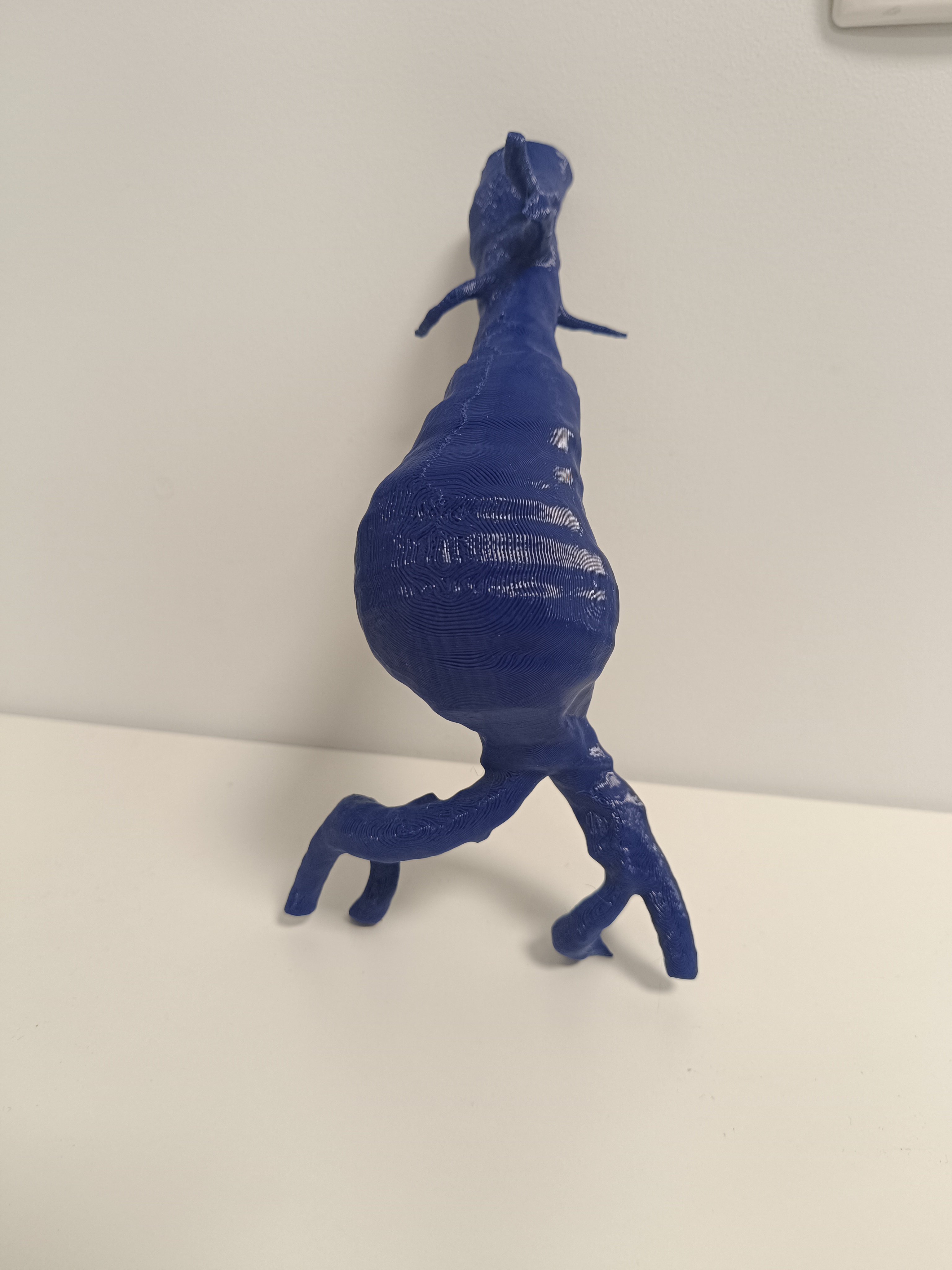

Supplement: Supplementary file 1 [file jcdd-11-00365-s001.zip › 1727619774195.jpg]

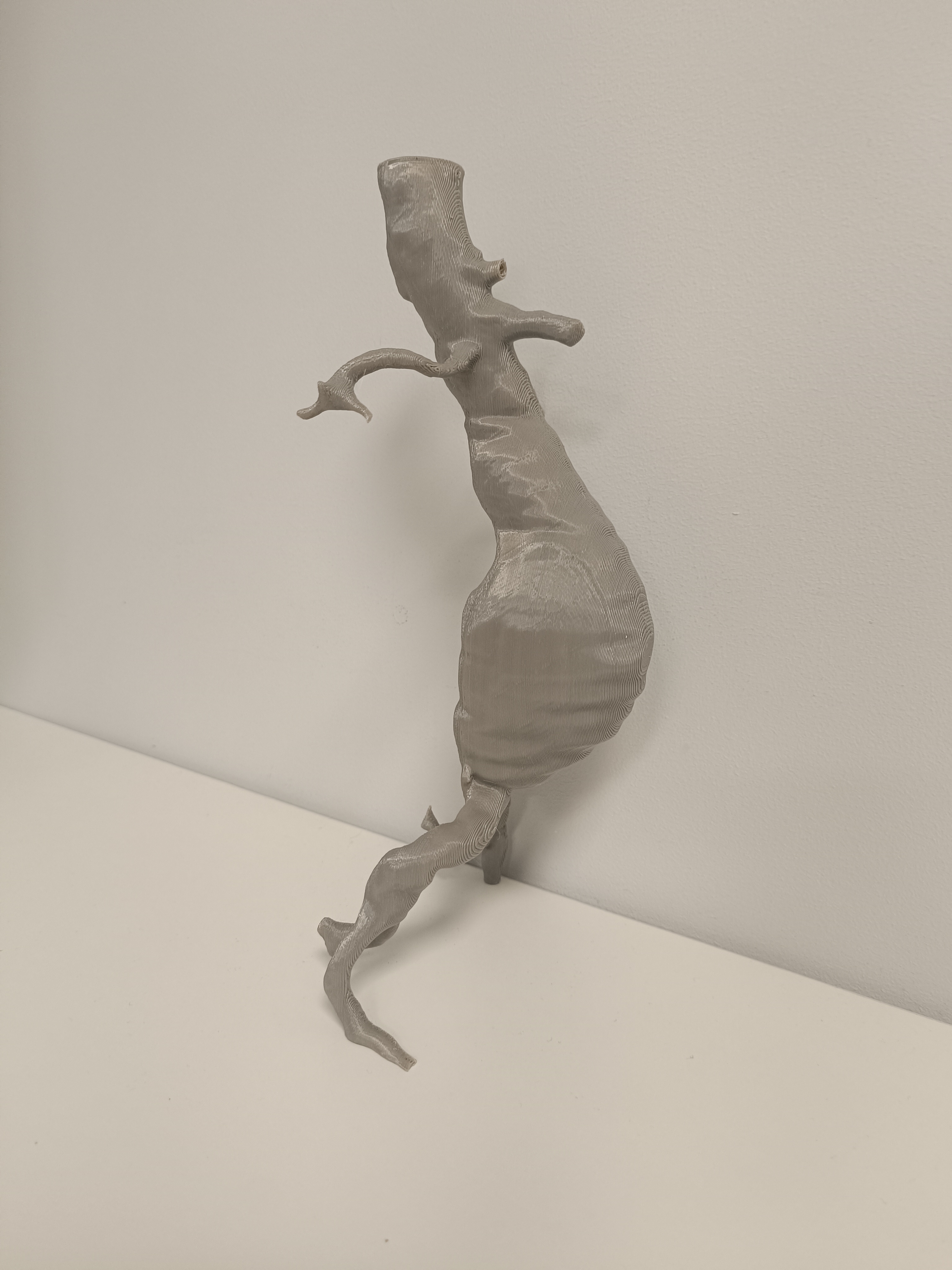

Supplement: Supplementary file 1 [file jcdd-11-00365-s001.zip › 1727619774181.jpg]

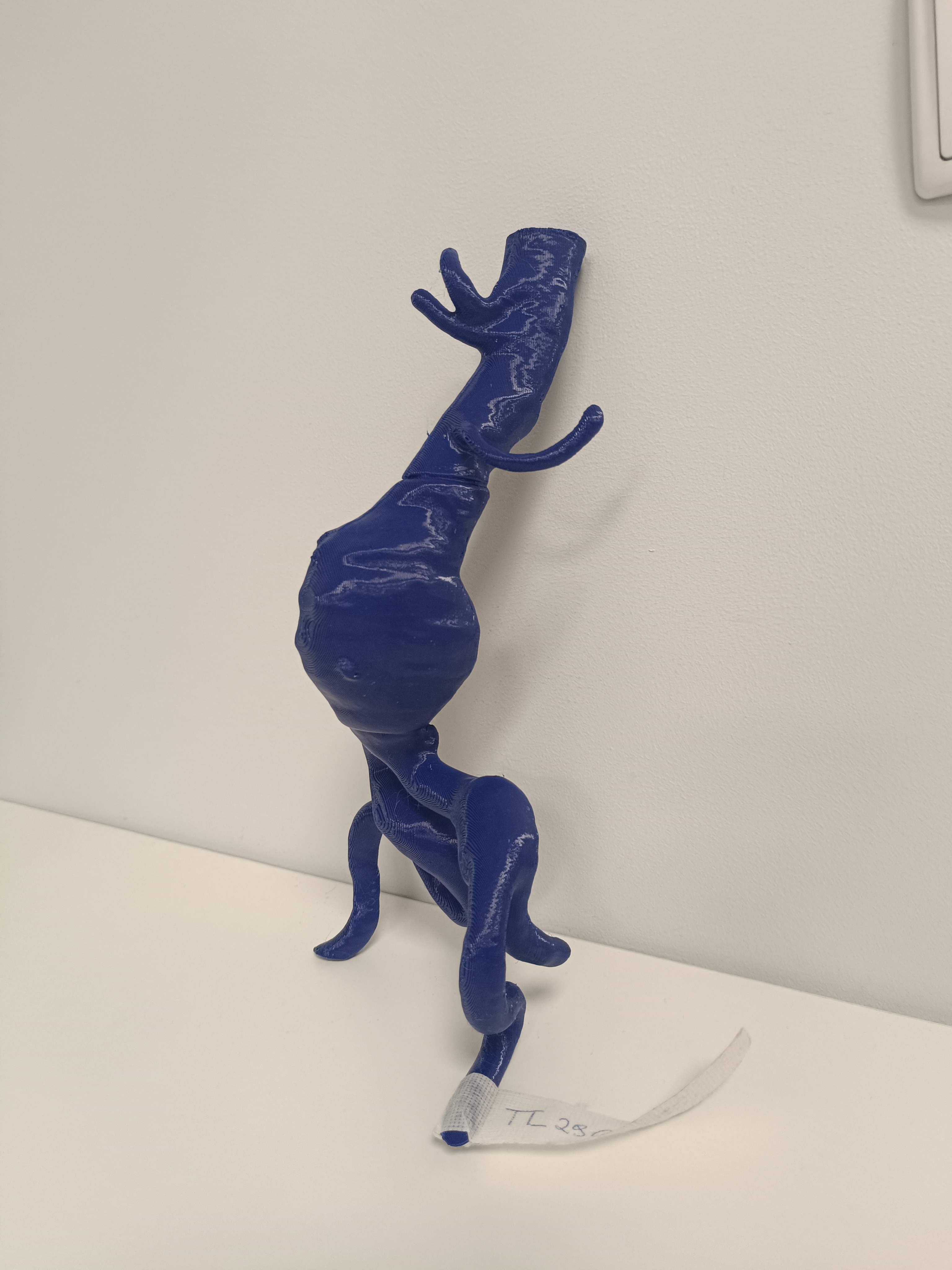

Supplement: Supplementary file 1 [file jcdd-11-00365-s001.zip › 1727619774174.jpg]
